# Integrative Approach to Address Male Infertility: A Case Study on Organophosphorus Compound Exposure and Traditional Medicinal Interventions

**DOI:** 10.7759/cureus.62697

**Published:** 2024-06-19

**Authors:** Priyansh Mathiya, Nancy Nair, Brijraj Singh, Akash More, Charu Pareek

**Affiliations:** 1 Clinical Embryology, Datta Meghe Institute of Higher Education and Research, Wardha, IND; 2 Anatomy, Datta Meghe Medical College, Datta Meghe Institute of Higher Education and Research, Nagpur, IND

**Keywords:** picsi, pseudocholinesterase, acetylcholinesterase, hypothalamic-pituitary-gonadal, organophosphorus compounds, endocrine-disrupting chemicals

## Abstract

Factors, including exposure to substances like organophosphorus compounds (OPCs), have been linked to fertility issues, which are a growing concern. In this case study, a 29-year-old farmer and his 26-year-old wife, married for the past five years, faced challenges conceiving despite several attempts. It was found that the husband's exposure to OPCs like chlorpyrifos, malathion, diazinon, etc., had impacted the quality of his sperm. However, after undergoing treatments and making lifestyle changes such as panchakarma therapy and taking Shilajit supplements, there was an improvement in sperm quality. Through in vitro fertilization using physiological intracytoplasmic sperm injection, successful fertilization and the development of high-quality blastocysts were achieved. This case demonstrates the potential for addressing infertility caused by toxins through a blend of traditional medicinal practices and modern reproductive technologies. It underscores the need for research into strategies that can reduce the effects of OPC exposure on male fertility.

## Introduction

Infertility is when a couple is unable to conceive after trying for a year without protection. It is estimated that around 17 to 20% of couples face difficulties in conceiving [1]. The presence of chemicals known as endocrine-disrupting chemicals (EDCs) has been linked to infertility, affecting aspects of reproductive health such as sperm production, quality, and the structure of the male reproductive system [2]. Studies indicate that exposure to EDCs can reduce sperm motility, concentration, and volume, cause abnormalities in sperm shape, and increase the likelihood of damage to sperm DNA. Male infertility can be caused by a variety of biological and environmental causes like exposure to toxins and pollutants, such as heavy metals, pesticides, and industrial chemicals, which can disrupt hormonal balance and impair reproductive function and also autoimmune diseases that attack sperm, diabetes, hormonal imbalances, genetic disorders, and other conditions. One such EDC that reduces male fertility is organophosphorus compounds (OPCs) [3]. Numerous environmental factors, such as pesticides and herbicides, have been linked to the global decline in male fertility. Exposure to agricultural chemicals such as OPCs, glyphosate, and atrazine has been associated with decreased sperm quality and fertility [4]. The most commonly cited toxins are believed to impact fertility through changes in the hypothalamic-pituitary-gonadal (HPG) axis or direct effects on sperm and other characteristics of semen. In pervasive environmental contamination, exposure to high concentrations of toxins may impact male fertility [5].

OPCs are potent neurotoxic chemicals characterized by their inhibitory action on acetylcholinesterase (AChE) enzymes, which causes abnormalities in sperm, including a decreased sperm count per ejaculation and a reduced percentage of viable sperm [6]. OPCs are readily absorbed by the cutaneous and respiratory epithelial membranes and dispersed, particularly in adipose tissues [6]. The majority of these OPCs are inactive when they are first noticed, but a process known as biotransformation allows them to become active. Phosphoric, phosphonic, and phosphinic acids are the precursors of OPCs. These substances belong to a broad class of organic substances mainly employed as insecticides. The most hazardous substances are utilized as nerve agents, while some are used as medications. AChE is a crucial enzyme in neurotransmission, and its suppression is the cause of the acute toxicity of organophosphorus chemicals [7].

Panic attacks, cramps, dizziness, headaches, nausea, skin rashes, and impaired vision are a few symptoms of these acute effects. On the other hand, the effects of prolonged exposure are marked by impact on reproduction, blood, nerve disorders, genetic changes, and congenital disabilities [8]. Large lymphocytes and abnormal cell topologies in the nuclear erythrocytes of blood cells are additional effects of these pesticides. Pesticide exposure also results in infertility and respiratory problems in people. OPCs cause cancer in addition to being mutagenic. Agricultural workers have shown significantly reduced serum luteinizing hormone and serum testosterone levels. OPC exposure disrupts the hypo-thalamic-pituitary endocrine function and indicates that follicle-stimulating hormone (FSH) and luteinizing hormone (LH) are the hormones most affected in males [9]. As part of the biomonitoring of exposure to OPC, butyrylcholinesterase (BChE) activity levels in biological samples are frequently measured. BChE is an enzyme that is present mainly in the liver and bloodstream. It is often referred to as plasma cholinesterase or pseudocholinesterase. It is essential to the metabolism of several substances, such as OPCs. The pseudocholinesterase test is relevant in OPC exposure cases due to its role in the metabolism and detoxification of certain OPCs [10]. OPCs are a class of chemicals commonly found in pesticides, insecticides, and nerve agents. They exert their toxic effects by inhibiting the activity of cholinesterase enzymes, including AChE and pseudocholinesterase. When an individual is exposed to OPCs, these substances can bind irreversibly to pseudocholinesterase, inhibiting its activity. As a result, the pseudocholinesterase levels in the blood decrease, which can be detected through laboratory testing. This reduction in pseudocholinesterase activity is a biomarker of exposure to OPCs [11].

To prevent injecting immature, anatomically defective spermatozoa, physiologically intracytoplasmic sperm injection (PICSI) selects mature sperm with an affinity for hyaluronic acid. Because only a mature sperm head can adhere to hyaluronan (HA), a crucial component of cumulus oophorus, and take part in the natural sperm selection activities, PICSI is used to select sperm. It is believed that sperm attached to hyaluronan exhibit increased maturity, reduced aneuploidies, acrosome activity, and sperm DNA fragmentation (SDF). It is, therefore, believed to enhance effective pre-implantation embryogenesis. As a sperm selection technique, PICSI is quite specific and poses few safety risks [12].

In Ayurveda, panchakarma therapies hold a special significance. It is mainly referred to as a body detoxification program. Panchkarma is necessary for the body to detoxify and for the embryo to have a healthy environment [13]. When it comes to female infertility, panchakarma, also known as ardhachikitsa (half treatment), serves as the foundation and support system for shaman chikitsa relief therapy. It cleanses the reproductive system and the entire body, giving expecting mothers both physical and mental well-being. It can also be viewed as a preconception therapy for a typical couple hoping for a safe and healthy pregnancy [14].

Indian subcontinental traditional healers refer to Shilajit as a vajikarak (aphrodisiac) and utilize it to cure male infertility as a result. Shilajit treats male infertility brought on by exposure to dangerous substances. Treatment with Shilajit increases the weight of the reproductive organs, the amount of sperm produced daily in the testicles, enzyme activities, and the level of testosterone in the serum [15].

## Case presentation

Patient information

A couple, with a wife aged 26 and a husband aged 29, came to our clinic after being married for four years, expressing concerns about their inability to conceive despite actively trying for three years. The husband, a farmer, had a history of smoking and alcohol or tobacco consumption, while neither partner had a history of thyroid issues, diabetes, or prior surgeries. The female partner, a homemaker, had no personal or family history of addiction, health problems, or genetic abnormalities. Since the female partner's menstrual cycle was regular and she had no known fertility issues, the problem was most likely with the male partner's reproductive health. He also reported a supportive and understanding relationship with his partner throughout the process and agreed to enroll for in vitro fertilization (IVF). Informed consent was obtained, and the patients were given a detailed explanation of all treatments and their limitations.

Medical history

The couple did not exhibit abnormalities and seemed to have good physical and social health, and his general health seemed to be expected. The patient denied any significant medical conditions, including sexually transmitted infections, urological disorders, or hormonal imbalances. The female had previously undergone two unsuccessful intrauterine insemination (IUI) cycles with one IVF cycle at several IVF facilities. The woman also disclosed a medical history of one IVF miscarriage. The present case of infertility was primary.

Clinical findings

The male patient seemed healthy and satiated. The patient's vital signs were within normal ranges. The patient had average muscle mass, a deep voice, and growing hair, all signs of secondary sexuality. There were no anomalies found during the genital examination. There was no discernible mass or soreness, and the testicles were reasonably sized and consistent. In 2022, the couple experienced two unsuccessful IUI attempts. Given that the female partner had regular menstrual cycles of ±28 days and had no history of conception issues, the issue was most likely related to the male partner's fertility. The male patient was advised for semen analysis and hormonal profile.

Diagnostic assessment

The male patient's seminal parameters were obtained: the sperm count was 10 million per milliliter (M/ml), and the sperm's total progressive motility was 20%, meaning the percentage of sperm that could move was determined. Eighty percent of the sample consists of nonmotile sperm, and the pH level indicates how acidic or alkaline the semen is. It was measured as 7.2 pH and 1.4 ml was the estimated ejaculate volume. The results of the morphological study raise severe concerns because just 2% of sperm have standard morphological traits, while 98% show abnormal morphology, which indicates structural defects. The patient was advised to undergo the SDF test and a sperm vitality test (Table 1).

**Table 1:** Semen analysis parameters before intervention. ml: Millimeter; pH: power of hydrogen; mil: millions

Semen parameters	Result	Reference value
Ejaculatory abstinence	Three days	2-7 days
Volume	1.4 ml	1.4-5 ml
Appearance	Grey opalescent	Grey opalescent
pH	7.2	7.2 -7.8
Sperm count	10 mil	≥16 mil
Total sperm motility	20%	40- 43%
Morphology	2 %	3.9 -4.0%

Table 1 describes the different parameters and their respective reference ranges and values. Oligoasthenoteratozoospermia (OATz) was indicated by the volume of 1.4 ml, sperm count of 10 million, total sperm motility of 20%, and morphology of 2%, all below the reference levels.

Hormonal parameters of the male partner are shown in Table 2, with the LH level higher at 8.0 mIU/ml. Progesterone, FSH, and prolactin levels were within normal ranges. However, testosterone levels were 1.78 ng/ml below the reference range. However at 58 pg/ml, estradiol levels were higher than the upper range.

**Table 2:** Hormonal profile of the male partner. LH: luteinizing hormone; FSH: follicle-stimulating hormone; mIU/mL: milli-international units per milliliter; ng/mL: nanograms per milliliter; pg/ml: picograms per milliliter

Parameters	Reference value	Result
LH mIU/mL	0.8-7.6 mIU/ml	8.0 mIU/ml
Testosterone ng/ml	2.50-9.50 ng/ml	1.78 mIU/ml
Progesterone ng/ml	0.27-0.9 ng/ml	0.7 ng/ml
Estradiol pg/ml	20-55 pg/ml	58 pg/ml
FSH mIU/ml	1.5-12.4 mIU/ml	1.22 mIU/ml
Prolactin ng/ml	<20 ng/ml	18 ng/ml

Therapeutic intervention

The patient was advised Shilajit 50 mg for 90 days and panchakarma; according to ayurvedic treatment guidelines, shodhan chikitsa performs panchakarma, or beginning management, which includes intra urethral uttar basti for a continuous period of 3-5 days per month for a maximum of 4-5 months. Shaman chikitsa, or medication treatment, is then advised for four months. Following four months of therapy, semen analysis was recommended, and the seminal parameter showed a notable improvement, with an increase in sperm count and motility as shown in Table 3.

**Table 3:** Semen parameters post intervention. pH: power of hydrogen

Semen parameters	Result	Reference value
Ejaculatory abstinence	Three days	2-7 days
Volume	1.5 ml	1.4-5 ml
Appearance	Grey opalescent	Grey opalescent
pH	7.2	7.2 -7.8
Sperm count	26 million/ml	≥16 million/ml
Total sperm motility	45%	40- 43%
Morphology	15 %	3.9 -4.0%

**Figure 1:**
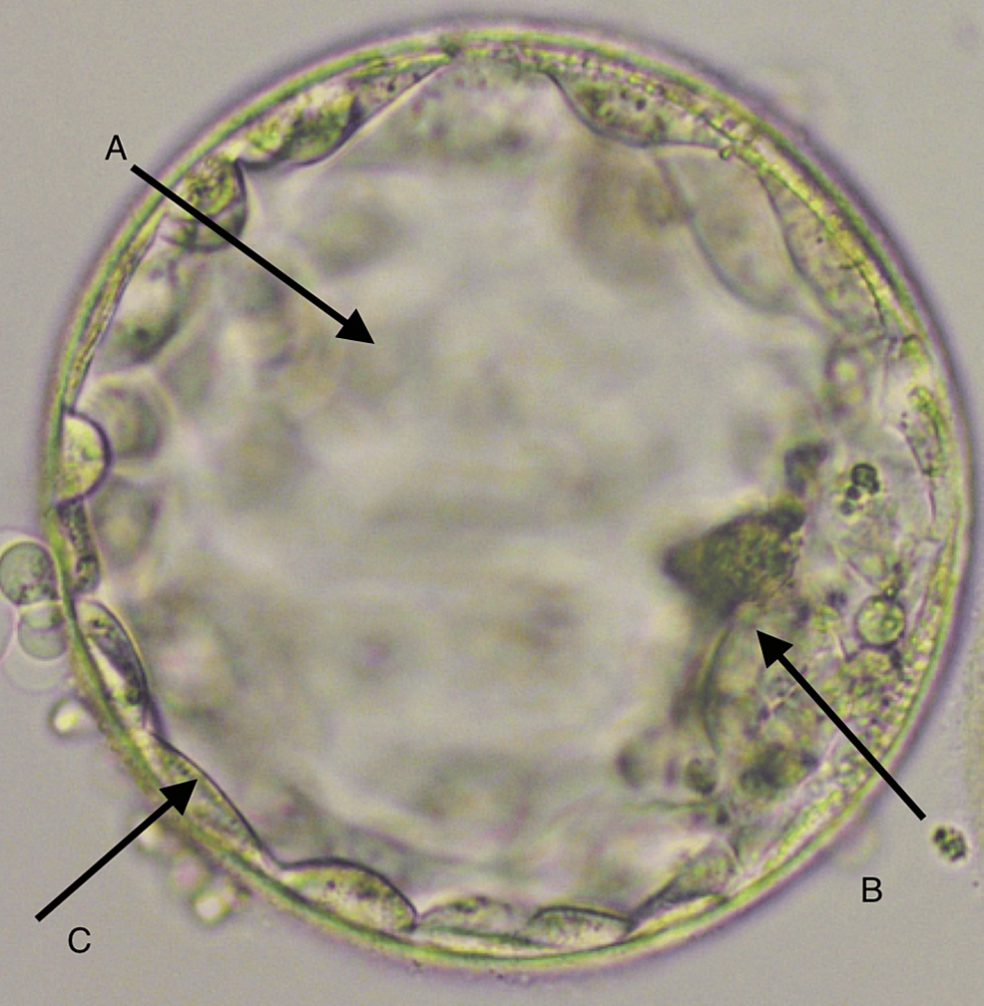
Day 5 blastocysts of grade 4BA selected for ET A: 4 size of the blastocyst (260 µm), B: inner cellular mass, A: trophectoderm, ET: embryo transfer

The patients signed an informed consent form before the stimulation regimen began. A gonadotropin-releasing hormone (GnRH) antagonist regimen was administered to the woman. For the first five days, she was given 150 IU of human recombinant follicle-stimulating hormone daily. The next three days, she received 150 IU of human menopausal gonadotropin and 0.25 mg of cetrorelix subcutaneously. Finally, on the 10th day, she was given 10,000 IU of recombinant human chorionic gonadotropin (hCG) and 0.2 mg of decapeptyl. Three and a half hours after the trigger was given, oocyte pick-up was carried out under transvaginal ultrasound guidance. A total of 11 oocytes were collected, of which four were in metaphase I (MI) and seven were in metaphase II (MII). After oocyte denudation, the oocytes were cultured in a fertilization medium till the following day. A fresh semen sample was used for the process. The semen sample was prepared using the density gradient method after PICSI post-sperm separation. The PICSI method was carried out according to protocol, which involved preparing the sperm sample, creating a PICSI plate with a droplet of HA to simulate sperm natural selection, and performing Intracytoplasmic sperm injection (ICSI) once sperm selection was successful. Retrieved spermatozoa were injected into each of these seven oocytes. The fertilized oocytes underwent successive cultures. The pronuclei were examined following 17 hours of ICSI to verify the success of fertilization. Two good-quality day 5 blastocysts of grades 3AA and 4BA, as per Gardaner's grading system, were produced from the seven fertilized oocytes, as shown in Figure 1.

Follow-up

Two good-quality day 5 blastocysts of grades 3AA and 4BA, as per Gardaner's grading system, were produced from the seven fertilized oocytes. Two months later, in the first cycle of frozen embryo transfer, a single frozen embryo of grade 4BA was chosen for the embryo transfer on day 12 of the ± 28 days of the menstrual cycle. The urine pregnancy test result was positive, and the level of beta-human chorionic gonadotropin (β-hCG) in the serum (973 mIU/ml) indicated a successful pregnancy.

## Discussion

This study highlights how exposure to OPCs can significantly affect male reproductive health, leading to issues such as decreased sperm quality as well as hormonal disruption. This raises concerns regarding the necessity for preventative actions and the broader effects of environmental pollutants on human fertility.

Mandi et al. examined the harmful consequences of long-term sub-lethal organophosphate exposure in the male reproductive system of a non-target model organism using morphological, biochemical, and behavioral characteristics [16]. Compared to their control counterparts, it was found that the physical, biochemical, and reproductive health of those exposed to the organophosphate chemical acephate over an extended period had been damaged. Male reproductive success varies during a person's lifespan depending on how many distinct features are expressed that are important for reproduction. Natural selection has been observed to favor characteristics that enable males to offspring, albeit trade-offs may impact how these traits manifest. Several studies concluded that environmental factors are vital in moderating these trade-offs [17,18]. Therefore, if males have limited energy, investing more in one reproductive trait may lead to less expression of another. The male drosophilids displayed decreased reproductive potential due to a change in resource allocation that helped them survive in the acephate-contaminated (stressed) environment [16].

A study by Sengupta et al. concluded that pesticide exposure significantly reduces sperm counts, much below the threshold for male fertility. There is large-scale research evaluating their links to human infertility, even though it is established that pesticide exposure is linked to infertility. It is still unclear how precisely male-mediated toxicity contributes to adverse outcomes, including abortions, congenital deformities, premature births, and so on [19]. The available information on several endocrine disruptors and risk factors, such as malnourishment and diseases that can increase the risk, points to a higher susceptibility of the populace in developing or impoverished nations to reproductive risks resulting from pesticide exposure [19]. For this reason, it is imperative to continuously track trends in semen quality within a particular demographic and ethnic group as well as exposure to and effects on parameters like congenital abnormalities, abnormal growth and development, testicular cancer, hypospadias, cryptorchidism, breast cancer, and so forth [19].

Positive results from the statistical study by Erberelli et al. demonstrated the effectiveness of the PICSI approach. The study found that the PICSI approach considerably increased the likelihood of conception in those who utilized it. The PICSI group had decreased abortion rates, but the study also demonstrates successful clinical pregnancies with live, healthy neonates that exhibit no abnormalities [20].

Kumar et al. stated that ksheena shukra dosha has an obvious treatment procedure in Ayurveda. In the instance of ksheena shukra dosha, they genuinely want the targeted shukravaha srotas, which is precisely what the particular shodhana therapy (virechana) hits on. Virechana karma aids in shukragni stimulation and srotorodha elimination. As a result, virechana karma speeds up the body's metabolism, increasing the amount and caliber of semen. Shodhan therapy enhances both physical and mental well-being by detoxifying the body. The patient received successful treatment, and no side effects were discovered. The course of treatment is also quite affordable [21].

A study by Biswas et al. reported that there was a noticeable improvement in the semen quality of the treated oligospermic individual, and this improvement was likely caused in part by the processed Shilajit (PS) substances in the semen. Four PS-treated patients who were able to conceive again provided hope for using natural products to treat oligospermia. However, it's still unclear how exactly these elements affect testicular function. If the PS treatment is administered for an extended time, this pattern should become more representative [22]. In our study also, an intriguing angle to the management is provided by the mention of ayurvedic treatments like panchakarma and the application of Shilajit to male infertility. Understanding the possible benefits of combining traditional healing methods with mainstream medical treatments could help us know holistic approaches to reproduction problems.

## Conclusions

This case study underscores the efficacy of amalgamating Ayurvedic interventions with the PICSI technique for augmenting sperm quality in individuals afflicted with male infertility attributed to OPCs exposure. The favorable outcome herein underscores the feasibility of integrating modern reproductive technologies with traditional therapeutic modalities to address complex reproductive challenges exacerbated by environmental pollutants. Moreover, it emphasizes the importance of comprehensive evaluation and personalized treatment regimens in the management of male infertility, particularly in contexts involving environmental exposures. Enhanced understanding and refinement of treatment protocols for analogous clinical scenarios necessitate further exploration of interventions aimed at mitigating the deleterious effects of OPC exposure on male fertility.
